# Identification of Pharmacological Targets in Amyotrophic Lateral Sclerosis Through Genomic Analysis of Deregulated Genes and Pathways

**DOI:** 10.2174/138920212800793366

**Published:** 2012-06

**Authors:** Sabrina Paratore, Salvatore Pezzino, Sebastiano Cavallaro

**Affiliations:** 1 Functional Genomics Center, Institute of Neurological Sciences, Italian National Research Council, Catania, Italy; 2 Policlinico-Vittorio Emanuele, University Hospital, Catania, Italy

**Keywords:** ALS, drug, pathway, pharmacogenomics, networks, target.

## Abstract

Amyotrophic Lateral Sclerosis (ALS) is a progressive and disabling neurodegenerative disorder characterized by upper and lower motor neuron loss, leading to respiratory insufficiency and death after 3-5 years. Riluzole is currently the only FDA approved drug for ALS, but it has only modest effects on survival. The majority of ALS cases are sporadic and probably associated to a multifactorial etiology. With the completion of genome sequencing in humans and model organisms, together with the advent of DNA microarray technology, the transcriptional cascades and networks underlying neurodegeneration in ALS are being elucidated providing new potential pharmacological targets. The main challenge now is the effective screening of the myriad of targets to identify those with the most therapeutic utility. The present review will illustrate how the identification, prioritization and validation of preclinical therapeutics can be achieved through genomic analysis of critical pathways and networks deregulated in ALS pathology.

## INTRODUCTION

Amyotrophic Lateral Sclerosis (ALS) is a progressive, disabling neurodegenerative disorder characterized by upper and lower motor neuron loss, leading to respiratory insufficiency and death after 3-5 years [1, 2]. The incidence of ALS ranges from 1.7 to 2.3 cases per 100,000 population per year world-wide [3]. Currently, ALS is an incurable disease and the only FDA approved drug, Riluzole, has very modest efficacy on survival [4].

Despite intensive research, knowledge of the pathogenetic mechanisms and precise genetic causes of ALS remains incomplete. Although most cases of ALS are sporadic (SALS), about 10% are familial (FALS), mostly with autosomal dominant inheritance [5]. In 3-7% of all ALS and 20% of FALS cases, different mutations in the gene encoding copper–zinc superoxide dismutase (*SOD1*) have been found [6]. In addition to SOD1, several mutations in other genes, including Senataxin (*SETX*) [7], Vesicle-associated associated protein B (*VAPB*) [8], and Alsin (*ALS2*) [9, 10], Spatacsin (*SPG11*) [11], Angiogenin (*ANG*) [12], PI(3,5)P(2)5-phosphatase (Fig. **4**) [13], and Optineurin (*OPTN*) [14] have been identified as causative for classical FALS. The etiology of SALS is still unknown but it is now widely accepted that SALS is a multi-factorial complex disease, which is attributable to and influenced by the interaction of environmental factors with multiple genes, such as those encoding for the heavy neurofilament subunit, peripherin, dynactin, and FLJ10986 [15]. An exciting step forward in ALS genetics is represented by the recent discovery of mutations in *TDP-43* (encoded by TARDBP) [16] and the related RNA-binding protein fused in sarcoma/translocated in liposarcoma (*FUS*) [17] in familial and sporadic cases that has shifted the focus of much research on RNA metabolism, and implicated abnormal RNA processing in ALS pathogenesis. Further advances in ALS etiology have also been obtained through the use of the new next-generation sequencing technology that allows to rapidly screen common genetic variation across the human genome [18]. A two-stage genome-wide association study (GWAS), which included data from nearly 20000 patients with sporadic ALS, has identified mutations in the *UNC13A* (unc-13 homolog A) gene and found a signiﬁcant association with the 9p21 chromosomal locus [19]. Importantly, the same chromosomal region has been confirmed in large independent GWASs of both ALS and Frontotemporal dementia (FTD), implicating the genetic defect at chromosome 9p in sporadic forms of both diseases [19-22]. Furthermore, by the same approach an expanded hexanucleotide repeat in a noncoding region of chromosome 9 open reading frame 72 (*C9ORF72*) has been identified as the most common cause of familial ALS, FTD and ALS-FTD forms [23, 24]. Despite common genetic variants are beginning to be unequivocally linked to ALS, to date its pathogenesis is not clearly understood. Additional works therefore are needed to better understand and effectively treat this disease. 

While the deep genome sequencing technologies are rapidly emerging as a useful method for the discovery of ALS susceptibility and causative genes, DNA-microarray is currently the most widely high throughput technology used to clarify the ALS pathogenic mechanisms. In the last ten years, genome-wide expression analysis by DNA microarray technology has been conducted on various tissues from rodent models [25-33] and ALS patients [34-36]. Our research group, in particular, has identified genes de-regulated in the motor cortex of patients with sporadic ALS, and interpreted the role of individual candidate genes in a framework of differentially expressed pathways [36]. Genomic-based studies are beginning to elucidate the transcriptional cascades and networks underlying neurodegeneration in ALS, drawing a precise molecular portrait of the pathology [37]. In addition to providing an unprecedented experimental opportunity to investigate ALS disease, gene expression profiling studies have also allowed the identification of new potential pharmacological targets. The main challenge is now the effective screening of the potential targets to identify those with the most therapeutic utility. 

**Fig. (1):**
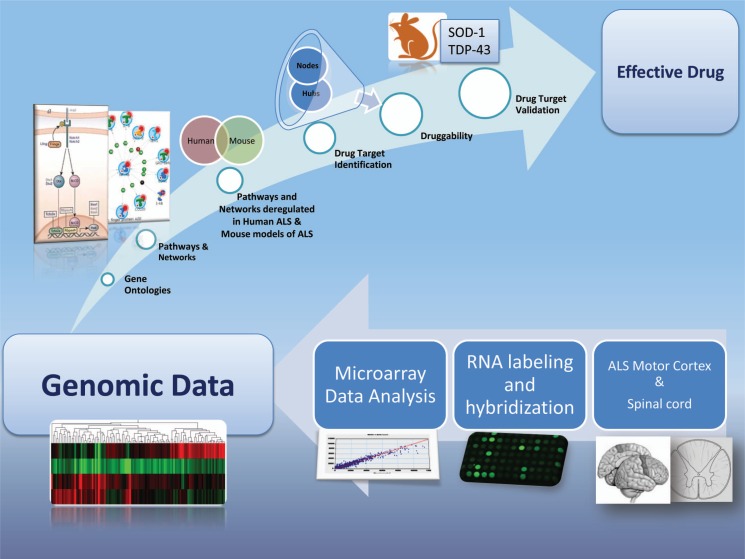
Genomic approach for drug discovery in ALS. A drug discovery pipeline starting from microarray analysis moves through drug targets identification and ends with their validation in animal models of ALS.

In the present review, we will illustrate how the identification, prioritization and validation of preclinical therapeutics can be achieved through microarray-based transcriptomic analysis of critical pathways and networks deregulated in ALS pathology (Fig. **1**).

## DIFFERENTIALLY EXPRESSED GENES RELEVANT TO ALS

**Fig. (2):**
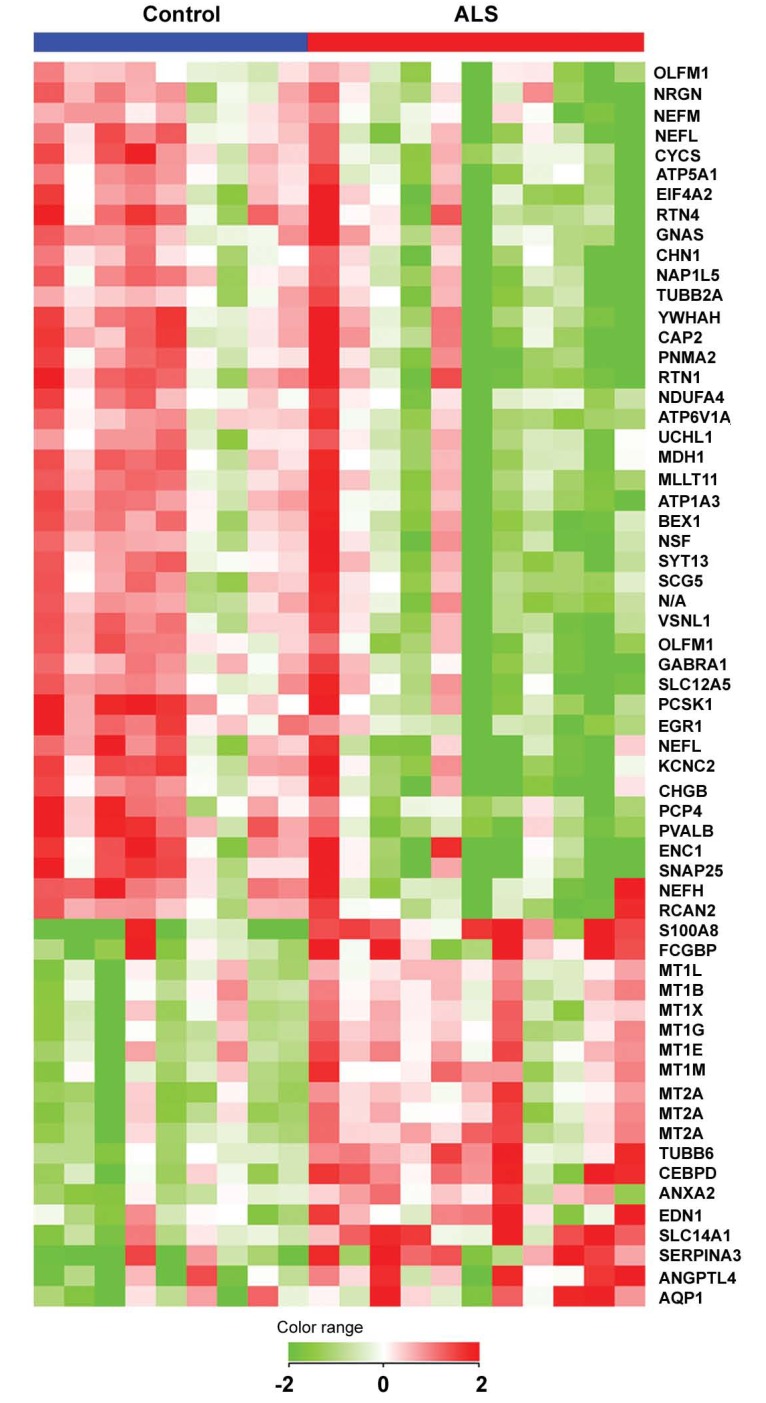
Hierarchical cluster of genes differentially expressed in motor cortex of SALS subjects. 57 of 19,431 quality-filtered genes (0.3%), represented by 61 probes, are differentially expressed, with each row in the matrix representing a single probe and each column a subject. Normalized expression levels are represented by the color of the corresponding cell, relative to the median abundance of each gene for each subject (see scale). Genes are named using their UniGene symbol and arranged in a hierarchical cluster (standard correlation) based on their expression patterns, combined with a dendrogram whose branch lengths reflect the relatedness of expression patterns.

As previously discussed, the etio-pathogenesis of sporadic ALS is largely unknown but it is now widely accepted that SALS is a multifactorial complex disease, which is attributable and influenced by the interaction of environmental factors and lifestyles with multiple genes. The sequencing of the human genome, and the development of high throughput technologies, offer today an unprecedented experimental opportunity to investigate ALS disease through a genomic approach. To illustrate this approach, we will refer to our previous published study where we examined whole genome expression profiles of motor cortex in control and sporadic ALS patient [36]. This screening revealed the differential expression of 57 genes (40 genes showing down-regulation and 17 up-regulation) (Fig. **2**), which may be involved in ALS neurodegeneration and offer potential pharmacological targets. To exemplify the use of single genes as potential pharmacological targets, below we will discuss the down regulation of four genes whose encoding proteins are involved with ion homeostasis and excitotoxicity: GABA-A receptor alpha1-subunit (*GABRA1*), ATPase - Na^+^/K^+^ transporting - alpha 3 polypeptide (*ATP1A3*), potassium voltage-gated channel subfamily C member 2 (*KCNC2*), and solute carrier family 12 (potassium/chloride transporter) - member 5 (*SLC12A5*). For a complete description of all the differentially expressed genes, the reader is referred to our previous study [36]. Reduced level of GABRA1 was previously demonstrated in human motor cortex of ALS patients and was associated with heightened excitability [38]. Mutations of the plasma-membrane Na^+^/K^+ ^pump ATP1A3 have been linked to rapid-onset dystonia parkinsonism [39] and its expression is reduced in SOD1(G93A) FALS mice [40] and in motoneurons of Wobbler mice [41]. *KCNC2* regulates the voltage-dependent potassium ion permeability of excitable membranes and its decreased expression may lead to decreased potassium conductance and delayed repolarization of axons [42]. *SLC12A5 *encodes the potassium-chloride cotransporter KCC2, a neuronal isoform of the potassium-chloride cotransporter family [43]. During early development, increased expression of *SLC12A5* lowers the intra-neuronal chloride concentration below its electrochemical equilibrium and allows GABA to act as an inhibitory neurotransmitter [44]. Conversely, a switch of GABA action from inhibitory to excitatory has been proposed as a mechanism contributing to excitotoxicity in injured neurons. Indeed, down-regulation of *SLC12A5* expression together with GABAA receptor-mediated excitation occurs after axonal or spinal cord injury [45, 46], and mouse *SLC12A5* knockouts suffer severe motor deficits and immediate postnatal death by asphyxiation [47].

As we have seen in the previous paragraph, microarray analysis allows identifying genes that are significantly differentially expressed in ALS. Although, some of their encoded proteins may be potential pharmacological targets, differentially expressed genes represent only the tip of the “iceberg” of a genomic analysis. When genes are analyzed individually, small changes in expression may not pass stringent statistical cut off. Those small changes; however, may show a statistical significance when analyzed, for example, in the context of a pathway or a network. In the following sections, we will illustrate how these types of analyses are of fundamental help to extract more knowledge and discover more complex relationships from genomic data.

## GENE ONTOLOGIES, PATHWAYS AND PROTEIN INTERACTING NETWORKS: THEIR IMPLICATION FOR DRUG TARGET DISCOVERY IN ALS

Cellular processes depend on the activity of an integrated network of genes and their encoded proteins, which almost never work alone but interact with one another in highly structured and incredibly complex ways. In this integrated network it is not important the activity of the single gene and their encoded protein, but the entire components and their interactions, a concept that can be summarized in: “*The whole is greater than the sum of its parts*”. Thus, genes do not act by themselves, but they function in gene networks and molecular pathways and their effects are not independent but often modified by one or several other genes (epistasis) [48]. Gene ontology enrichment and protein interacting networks of microarray data allow us to look at the “whole” instead of the “single parts”. 

### Gene Ontology Enrichment

**Fig. (3):**
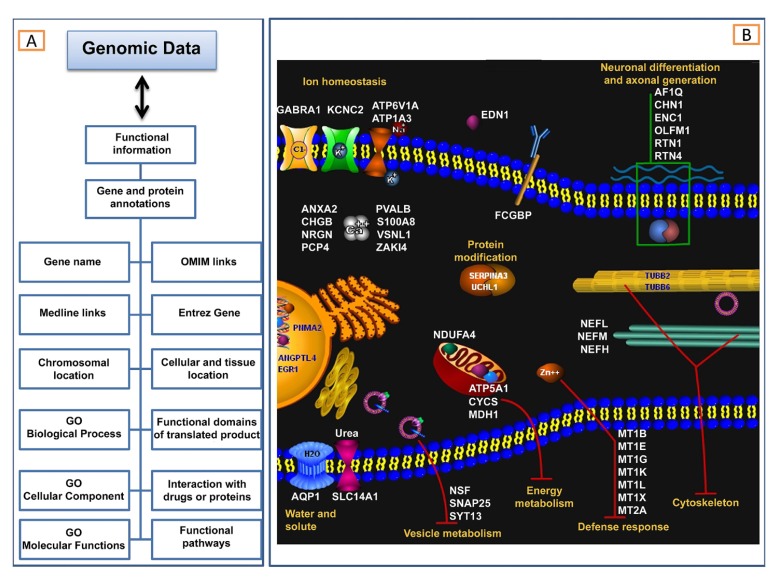
Gene ontologies. (**A**) Correlations between microarray data and functional information. Complex correlations between gene expression profiles and functional annotations are needed to extract more knowledge. Examples of functional information are listed on the left side of the figure and include gene or protein annotations. (**B**) GO analysis of genes differently expressed in cortex of sporadic ALS patients reveals the involvement of specific cellular processes and sub-cellular compartments.

**Fig. (4):**
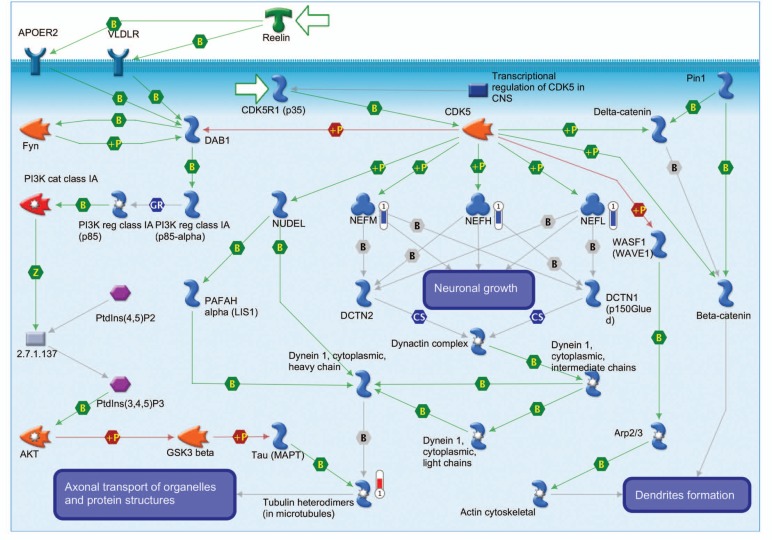
Cytoskeleton remodeling signaling, an example of a differentially regulated pathway in the motor cortex of SALS. Thermometers labeled with (1) or (2) indicate expression levels in motor cortex and spinal cord, respectively. Downward thermometers have blue color and indicate down-regulated expression, whereas upward thermometers have red color and indicate up-regulated expression. The mechanism of physical interaction is indicated: B, binding; +P, phosphorylation; T, transformation; TR, transcription regulation; Z, catalysis.

A gene or its encoded protein has not only a name/symbol or an expression value, but also several ontologies or functional annotations (Fig. **3A**).

Common functional annotations are those listed in the Gene Ontology (GO) database (www.geneontology.org), a controlled vocabulary of terms that describes the roles of genes and proteins in all organisms [49]. GO is comprised of three independent ontologies: 1) *biological process *describes biological goals accomplished by one or more ordered assemblies of molecular functions; 2) *cellular component *describes locations, at the levels of sub cellular structures and macromolecular complexes; 3) *molecular function *describes activities, such as catalytic or binding activities, at the molecular level. Biological process, molecular function and cellular component are all attributes of genes, gene products or gene-product groups and each of these may be assigned independently. The relationships between a gene product (or a gene-product group) to biological process, molecular function and cellular component are one-to-many, reflecting the biological reality that a particular protein may function in several processes, contain domains that carry out diverse molecular functions, and participate in multiple alternative interactions with other proteins, organelles or locations in the cell. Through the use of GO terms, a number of software tools (www.geneontology.org/GO.tools.shtml) are able to perform gene ontology enrichment analysis of high-throughput experimental results, such as gene expression microarray data, and discover statistically significantly enriched GO terms among a given gene list. An example of gene ontology enrichment based on GO terms is shown in Fig. (**3B**), where GO analysis of genes differently expressed in cortex of sporadic ALS patients reveals the involvement of specific cellular processes and sub-cellular compartments.

### Pathways Analysis

In addition to the GO terms described above, many other annotations are nowadays linked to a specific gene/protein. Examples of these are the associated disease (OMIM Links), publications (Medline links), chromosomal location, interacting drug, functional domain, and functional pathway (Fig. **3A**). This last, in particular, represents a set of consecutive signals or metabolic transformations that have been confirmed as a whole by experimental data. Thousand of pathways are nowadays available (for a list of biological pathway related resources see: www.pathguide.org) and different informatics tools have been developed that enable to analyze gene expression changes in the context of pathways. Below we describe some of these public and private resources. The Kyoto Encyclopedia of Genes and Genomes (KEGG) [50] is a free resource that contains a comprehensive collection of databases for genes, pathways and ligands for several organisms, together with web-accessible tools for the retrieval of pathways and the annotation of gene lists. The Gene Map Annotator and Pathway Profiler (GenMAPP) [51] are freely available programs for viewing and analyzing gene expression data in the context of biological pathways. Examples of private resources include MetaCore (www.genego.com), Ingenuity Pathways Analysis (www.ingenuity.com), Pathway Assist (www.ariadnegenomics.com) and GeneSpring (www.agilent.com).

Pathway analysis of genes differentially expressed in motor cortex of ALS patients has been described in more detail in our previous study [36]. In the following paragraph and in Fig. (**4**), we describe an example of a pathway differentially affected in ALS, the Cytoskeleton remodeling signaling.

The cytoskeleton is critical for neuronal maintenance and plasticity, neurite outgrowth, axonal calibre and transport. As illustrated in Fig. (**4**), our pathway-based analysis in motor cortex of SALS patient reveals the alteration of two major components of the neuronal cytoskeleton in the ALS motor cortex, showing a general down-regulation of microtubules and deregulation of genes encoding tubulin proteins (Tubulin heterodimers), as well as decreased expression of all three neurofilament subunits (*NEFM*, *NEFL*, *NEFH*). A depletion of microtubules and neurofilaments has deleterious effects on motoneurons, according to our understanding of their role in ALS pathogenesis [52]. Impaired microtubule-based axonal transport causes related motoneuropathies and is the earliest detectable, presymptomatic abnormality in SOD1-mutant FALS mice. In addition to this, defects in microtubule-associated motor proteins cause ALS-related human motoneuropathies, such as Charcot-Marie-Tooth disease [53] and hereditary spastic paraplegia [54], and are responsible for ALS phenotypes in Drosophila [55] and mouse [56]. The role of neurofilaments (NFs) in ALS is still controversial, despite a substantial body of research addressing the subject [52]. Deletion of the *NEFL* subunits in the SOD1 G85R mouse model is accompanied by preferential increase of the *NEFH* and *NEFM* subunits in the motor neuron cell bodies and reduction of these subunits in the axons, with an overall significant delay in the onset and progression of clinical disease [57]. Over-expression of the *NEFH *subunit has similar effects [58], prompting the hypothesis that NFs primarily act as an abundant buffer for otherwise deleterious processes, such as offering phosphorylation sites for deregulated intracellular kinases, or reducing the burden of axonal transport [52, 59, 60]. Furthermore, decreasing the axonal burden of neurofilaments may protect motor neurons, at least in part, by enhancing axonal transport, a hypothesis supported by the observation of defects in slow axonal transport in presymptomatic mutant SOD1 mice [61]. The down-regulation of all three NF subunits observed in our study would be detrimental according to either interpretation of NF involvement in ALS pathology, first by interfering with their stoichiometric balance, and second by depleting their availability as a potential buffer for aberrant enzymatic activities.

The above described example, other than visualizing gene expression changes in the context of a pre-drawn pathway, allows us to obtain a more complete and comprehensive view of ALS pathogenic mechanisms, providing a rational approach for drug target selection in ALS. Topics relating to drug selection, druggability and drug target validation will be discussed in the next paragraphs.

### Network Analysis and Druggability of Nodes and Hubs

Gene and protein-protein interactions are fundamental to all biological processes. A comprehensive determination of all interactions (*interactome*) [62] that can take place in an organism provides a framework for understanding biology as an integrated system (*system biology*) [63]. Network analysis has become a fundamental component of systems biology. Because such analysis provides a unifying language to describe relations within complex systems, it has assumed an increasingly significant role in understanding physiological and pathophysiological functions. Discovering the dynamic nature of cellular networks has relevance to human health, since defects in signaling and regulatory pathways are associated with many diseases, such as neurodegenerative disorders [64, 65]. In view of the gene and protein signaling networks associated to a complex disorder such as ALS, we may need to rethink our strategies for drug development, targeting ALS pathogenesis as a system rather than on the level of the single protein molecule. The current trend in pharmaceutical drug development is characterized by a re-evaluation of the “*one disease-one drug target*” paradigm that has dominated thinking in the pharmaceutical industry for the last few decades [66]. It is now widely accepted that many compounds do not exert their effects through a single target, instead they have multiple targets. In fact, drugs produce perturbations in a molecular system and very often modulate multiple target proteins. 

A network can be graphically represented by nodes (genes, proteins) connected by edges (nature of the interaction) and hubs (nodes connected to relatively many other nodes). There are several computational tools and protein-protein interaction database that can help to construct networks from high-dimensional biological datasets, such as microarray data. Some of these computational tools/protein-protein interaction databases are reported in Fig. (**5**) [2, 67-71]. In the next paragraph, we describe an example of a network of proteins encoded by differently expressed genes in SALS motor cortex, the Metallothioneins network.

As illustrated in Fig. (**6**), our previous genomic analysis in SALS patients reveals the coordinated and massive up-regulation of seven different metallothioneins (*MT1E*, *MT1G*, *MT1L*, *MT1M*, *MT1X*, *MT1B *and *MT1*). Metallothioneins (MTs) are low molecular weight, metal-binding proteins that act as important regulators of metal homeostasis (zinc and copper homeostasis), and as a source of zinc for incorporation into proteins, including zinc-dependent transcription factors [72]. Metallothioneins of the MT1 and MT2 families are able to prevent zinc deficiency *in vivo* [73], and have also been proposed to function as detoxifiers of heavy metals (zinc, cadmium, inorganic mercury and selenium) and free radicals [74, 75]. In addition to this, MTs seem to have a protective role in the CNS. Indeed, MT-1 and -2 increase in spinal cord of ALS patients and in transgenic mutant-SOD1 mice, where their experimental reduction significantly reduces survival. Importantly, endogenous MT1 and MT2 are undetectable in pure motoneuron cultures, which can be protected against oxidative stress by experimental MT1 over-expression [76], indicating the importance of metallothionein-mediated protection that is normally provided by astrocytes. Furthermore, exogenous MT1 and MT2 uptake promotes axon regeneration *in vitro* and *in vivo*, suggesting their potential as therapeutic agents [77].

Once identified, deregulated networks nodes and hubs can be topologically investigated for their gene expression pattern and their “druggability”, or else the ability of these objects to be targets for drugs currently on the market or molecules in the development pipeline [78-80]. Fig. (**7**) shows some bioinformatics and cheminformatics resources [50, 69, 81-85] that combine detailed drug data with comprehensive drug target information and allow investigating the targets druggability. As represented on Fig. (**6**) for the Metallothioneins network, a list of interacting drug-targets can be identified trough the use of these resources. Some drugs directly interact with their targets regulating their activities, whereas other drugs may interfere with target expression levels. For instance, the Cilostazol, an antiplatelet drug used for the treatment of intermittent claudication, may significantly increase MT protein levels in human neurons as an anti-oxidative effecter molecule [86, 87].

## DRUG TARGET VALIDATION

Once drug targets are established, they need to be validated. As we will discuss in the next section, this target validation consists in the accurate evaluation that a specific target is critically involved in a disease process and that modulates the target, is likely to have a desired therapeutic effect. The drug validation process relies on the use of different animal species as models of safety and efficacy before a new compound is administered to humans. Target validation studies are often conducted in mice due to their relatively small size, short generation times and the existence of capabilities such as gene knockout technology, which can be seen as analogous to antagonist treatment. All these experiments assume that modulation of a drug target will have a similar effect on the model species, as it would do on humans. Indeed, since the biology of the drug target differs across species and animal studies do not always translate successfully to humans, attrition rates in drug discovery remain very high [88]. In ALS, target validation is very often performed in rodent models that, as anticipated in the introduction, are monogenic and do not fully represent the complexity seen in human pathology. SALS is a polygenic and multifactorial disease and the utility of these animal models in the preclinical phase of pharmacological trials has been doubted. With these limitations, however, the use of these models is inevitable. Some established rodent models of ALS are listed in Fig. (**8**). Among these, the recent models based on (mutant) TDP-43 and FUS/TLS lack specificity in relation to the mutation and/or the cell type affected and are not yet used for routine drug screening [89-91]. The Superoxide dismutase-1 (SOD1) transgenic mice remain the most commonly used model for preclinical pharmacological trials [92-96]. Whole-genome expression analysis has been performed in some of these animal models, and deregulated genes or pathways are available in genomic database for further studies [97, 98]. The comparison of published gene expression data from human SALS and mouse ALS models can help researchers prioritize drug candidates for validation processes. The selection of common genomic changes between human and different ALS models is therefore feasible, and represents a possible strategy to mitigate the currently high attrition rate of pharmacological compounds for ALS.

## CONCLUSIONS

With the completion of genome sequencing in humans and model organisms, together with the advent of high-throughput technologies, the transcriptional cascades and networks deregulated in ALS are being elucidated providing new potential pharmacological targets [36]. The computational techniques associated with enormous datasets of information derived by high-throughput technologies, will allow to portrait the altered networks of biological molecules in ALS and provide important insights into drug targets capable of interfering with ALS pathogenesis [99]. In view of the gene- and protein-signaling networks associated to complex disorder such as ALS, we have to rethink our strategies for drug development, targeting ALS pathogenesis as a system rather than at the level of the single protein molecule.

## Figures and Tables

**Fig. (5):**
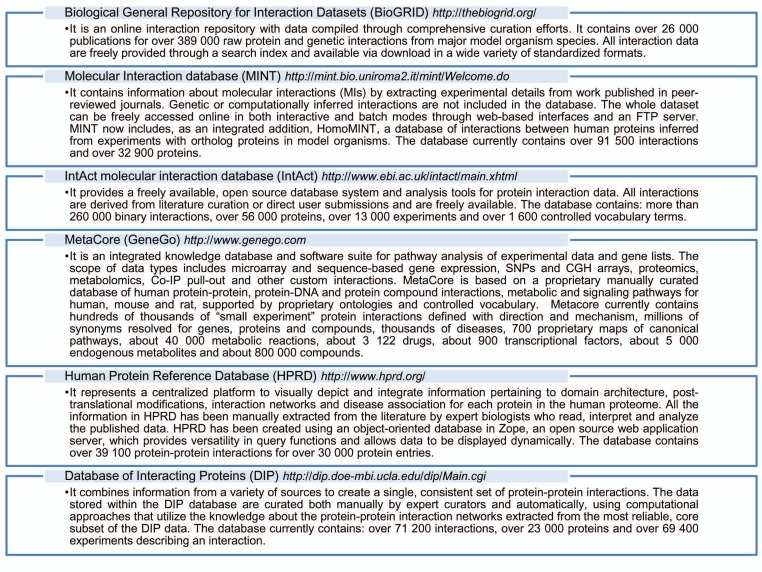
Several computational tools/protein-protein interaction databases.

**Fig. (6):**
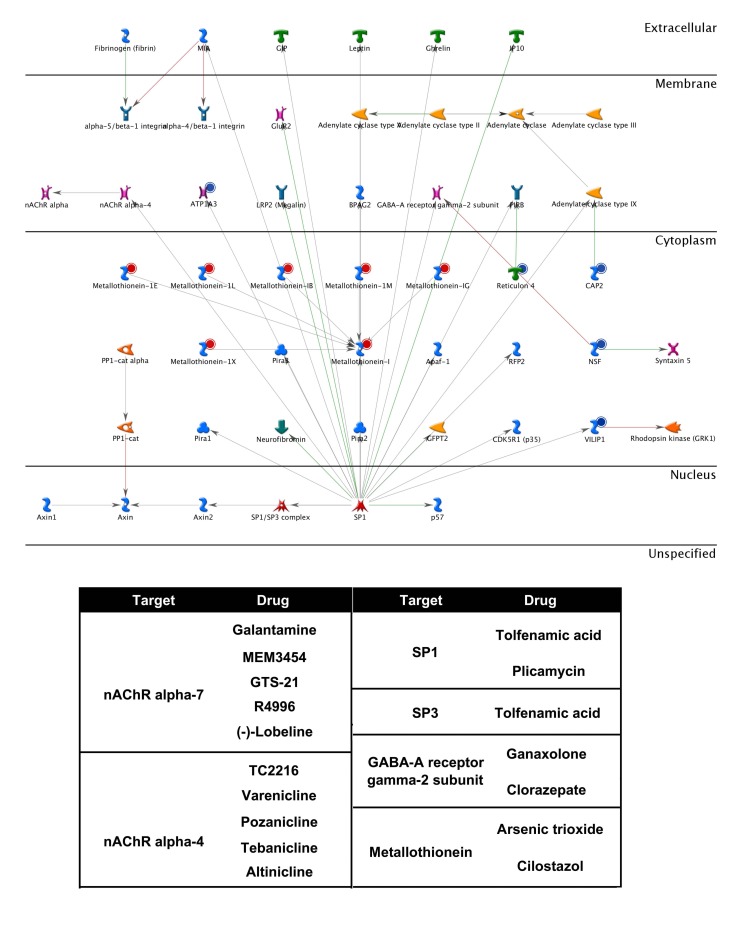
The Metallothioneins signaling network. Genes differentially expressed in motor cortex of sporadic ALS patients were uploaded into the MetaCore web portal (GeneGo) and their translated products (objects) used as the input list for generation of biological networks by the Analyze network algorithm with default settings. Up-regulated genes are marked with red circles, down regulated with blue circles; the green and red lines represent the activation or inhibition of the pathway, respectively. The network algorithm starts with building a “large network” by expanding the initial list of objects. Then, the large network is “cut” into smaller sub-networks highly saturated with objects of the input list, ranked by P-value and interpreted in terms of Gene Ontologies. The different objects (proteins, enzymes, receptors, channels, ligands) identified as pharmacological targets in the biological network, together with their drugs, are reported in the table at the bottom of the figure.

**Fig. (7):**
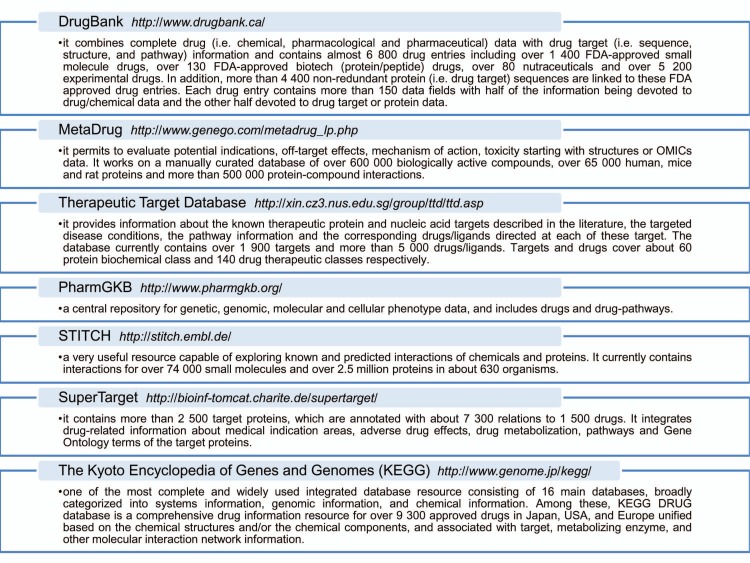
Example of some drug-target repositories.

**Fig. (8):**
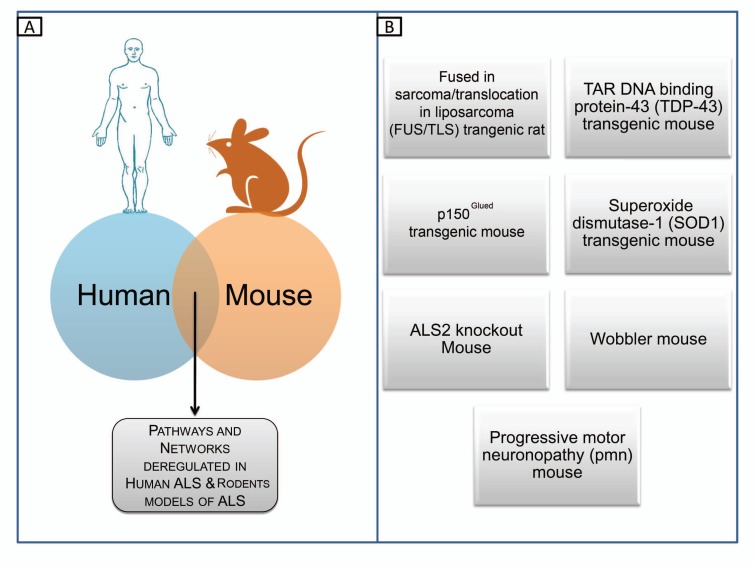
Drug target validation in mouse models of ALS. (**A**) Comparison between whole genome expression profiles of human SALS and mouse ALS models can provide the identification of common genomic changes. (**B**) Some established animal models of ALS.
